# A practical method for evaluating the *in vivo* efficacy of EVA-71 vaccine using a hSCARB2 knock-in mouse model

**DOI:** 10.1080/22221751.2021.1934558

**Published:** 2021-06-13

**Authors:** Yong Wu, Zhe Qu, Rui Xiong, Yanwei Yang, Susu Liu, Jianhui Nie, Chunnan Liang, Weijin Huang, Youchun Wang, Changfa Fan

**Affiliations:** a Division of Animal Model Research, Institute for Laboratory Animal Resources, National Institutes for Food and Drug Control (NIFDC), Beijing, People’s Republic of China; b National Center for Safety Evaluation of Drugs, Institute for Food and Drug Safety Evaluation, National Institutes for Food and Drug Control (NIFDC), Beijing, People’s Republic of China; c Division of HIV/AIDS and Sexually Transmitted Virus Vaccines, Institute for Biological Product Control, National Institutes for Food and Drug Control (NIFDC), Beijing, People’s Republic of China

**Keywords:** HFMD, hSCARB2 knock-in mouse model, *in vivo* vaccine efficacy, practical evaluation method, quality control

## Abstract

Hand-foot-and-mouth disease is a contagious disease common among children under 5 years old worldwide. It is caused by strains of enterovirus, especially EV-A71, which can lead to severe disease. Vaccines are the only way to fight this disease. Accordingly, it is necessary to establish an efficient and accurate methodology to evaluate vaccine efficacy *in vivo*. Here, we established a practical method using a hSCARB2 knock-in mouse model, which was susceptible to EV-A71 infection at 5–6 weeks of age, to directly determine the efficacy of vaccines. Unlike traditional approaches, one-week-old hSCARB2 mice were immunized twice with a licensed vaccine, with an interval of a week. The titre of antibodies was measured after 1 week. Mice at 4 weeks of age were challenged with EV-A71 intraperitoneally and intracranially, respectively. The unimmunized hSCARB2 mice displayed systemic clinical symptoms and succumbed to the disease at a rate of approximately 50%. High viral loads were detected in the lungs, brain, and muscles, accompanied by clear pathological changes. The expression of IL-1β, IL-13, IL-17, and TNF-α was significantly upregulated. By contrast, the immunized group was practically normal and indistinguishable from the control mice. These results indicate that the hSCARB2 knock-in mouse is susceptible to infection in adulthood, and the *in vivo* efficacy of EV-A71 vaccine could be directly evaluated in this mouse model. The method developed here may be used in the development of new vaccines against HFMD or quality control of licensed vaccines.

## Introduction

Hand-foot-and-mouth disease (HFMD) is a prominent epidemic disease in the Asia-Pacific region. It often exhibits seasonal increases in morbidity and mortality, especially in children under the age of five [1]. Enterovirus A71 (EV-A71), inclusive of coxsackievirus A10 (CVA10) and coxsackievirus A16 (CVA16), are the major etiological agents which belong to members of Enterovirus A species of the Enterovirus genus under the *Picornaviridae* family [2–4]. EV-A71 can cause severe neurological complications such as central nervous system symptoms due to its intrinsic neurotropism [5], whereby severe cases may result in convulsions, shock and even death [6]. In China, it was listed as a class C infectious disease by the ministry of health for management in 2008 [7], and the incidence of HFMD was ranked first [8,9].

Because there is still no specific therapeutic drug, vaccination is the only way to prevent and control this disease [10]. The successful development of a vaccine has played a great role in the control of HFMD caused by EV-A71, reducing the number of deaths from 142 in 2016 to 14 in 2019 [11]. At present, three inactivated EV-A71 vaccines are available in China, and the annual authorization quantity exceeds 10 million [12–15]. Some companies and academic institutions are still active in the research and development of novel types of vaccines [16–18] or polyvalent vaccines [19].

The *in vivo* protection efficacy of vaccines is of great concern to manufacturers, regulators, and consumers. Animal models that are susceptible to the virus are essential for assessing the protective effect of a vaccine [20,21]. Both non-human primate and mouse models of EV-A71 infection have been developed [2,22–24]. Due to economic and ethical considerations, mouse models are more widely used, but it was reported that wild-type inbred mice are optimally susceptible to EV-A71 only within 7 days of age, and mice older than 2 weeks were no longer susceptible [25,26]. Consequently, suckling mice under 5 days of age are usually selected for testing [10].

However, neonatal mice generally have a poor antibody response, and only animals aged 4–6 weeks can be selected for vaccination. Moreover, the production of neutralizing antibodies requires 2–3 weeks after primary immunization. This means that mice will be as old as 6–9 weeks of age when enough neutralizing antibodies were elicited, at which point they are no longer susceptible for enterovirus infection, and therefore no longer suited to evaluate the *in vivo* efficacy of HFMD vaccines [27]. To overcome this dilemma, an evaluation assay depending on maternally transferred antibodies was developed [28,29]. After immunizing the dams prior to gestation, the antibodies produced by the mother are transmitted to the suckling mice through the placenta, and the suckling mice are then challenged with EV-A71. The *in vivo* protective efficacy of the vaccine was calculated by comparing the clinical symptoms with those of suckling mice from unimmunized dams [30]. This assay process is tedious, and each suckling mouse may obtain a different amount of transferred maternal antibodies, resulting in irreproducible results.

Human scavenger receptor class B member 2 (hSCARB2) is widely expressed in many human tissues and cell types, such as lungs, muscles, and neurons [31,32]. Several studies have shown that hSCARB2 is the key receptor for EV-A71 infection in humans, both *in vitro* and *in vivo* [33–35]. Although the scavenger receptor class B member 2 of mice share significant sequence similarity with its human ortholog, it cannot mediate EV-A71 infection [36]. Early studies relied on transgenic mice expressing the hSCARB2 receptor [37–39]. A knock-in mouse model with a pure C57BL/6 background was constructed using embryonic stem-cell targeting technology in our own laboratory, and named the *C57BL/6-hSCARB2*^tm1(ROSA261^/NIFDC (short for hSCARB2) mouse [40]. As expected, the hSCARB2 mice were found to be more susceptible to infection with different EV-A71 strains, up to 5–6 weeks of age [41].

After obtaining this highly susceptible knock-in model, we envisioned that the maternal vaccination process may not be required when using the hSCARB2 mice as a model for testing vaccination efficacy against EV-A71 infection. Generally, at least two doses of a vaccine are needed to obtain high titres of neutralizing antibodies, with an interval of 1 week, and hSCARB2 mice are most susceptible at 3–4 weeks of age [41]. Accordingly, we inferred that the optimal time for the first vaccination would be at 1 week of age. Different from the traditional approaches, we selected one-week-old hSCARB2 mice, and not 4–6 week-old mice, to initiate vaccination with a marketed vaccine, after which binding and neutralizing antibodies were measured after an interval of 1 week. One week after the second immunization, the mice were challenged with EV-A71 either intraperitoneally or intracranially. The immunized mice generated high titres of both binding and neutralizing antibodies, and were effectively protected from a fatal virus challenge. By contrast, the unimmunized hSCARB2 mice displayed systemic clinical symptoms, with high viral loads in the lungs, brain and muscles, as well as pathological changes in several organs, leading to death. The expression of IL-3, IL-1β, IL-13, IL-17, and TNF-α was significantly upregulated.

## Materials and methods

### Animals and ethical review

The design of the experiment was approved by the ethics committee of the National Institute for Food and Drug Control (NIFDC). The development of the hSCARB2 knock-in mouse model has been described previously [40]. Both the hSCARB2 model and wild-type C57BL/6 mice were supplied by the Institute for Laboratory Animal Resources and were raised at the barrier facility of NIFDC.

### Immunization

**Figure 1.**
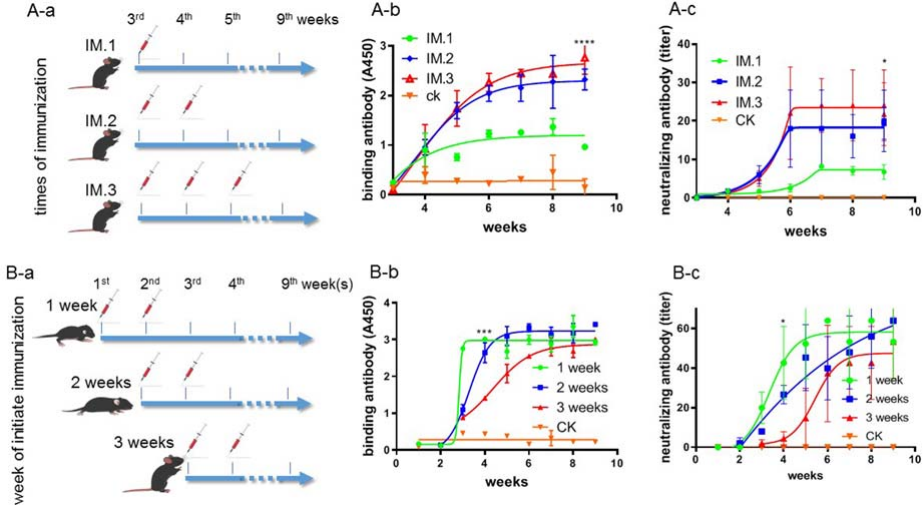
The immunization strategy of hSCARB2 mice and characterization of antibodies generated upon immunization. (A) Generation of antibodies after different repeated immunizations. The mice were immunized one, two, and three times from 3 weeks of age, with an interval of 1 week (*N* = 5). (A-b) The generation of binding antibodies after immunization, (A-c) the generation of neutralizing antibodies after immunization. Statistical analysis was conducted after the antibodies reached the plateau stage after immunization. (B) The generation of antibodies at different initial age at immunization. The mice were immunized two times from 1, 2, or 3 weeks of age, with an interval of 1 week (*N* = 5). (B-b) The generation of binding antibodies after immunization, (B-c) the generation of neutralizing antibodies after immunization. Statistical analysis was conducted at the age of 4 and 9 weeks. One-way ANOVA test was used for statistical analysis and the error bar of each group was included *P < .05, ***P < .001, and ****P < .0001.

An inactivated EV-A71 vaccine (Institute of Medical Biology, Chinese Academy of Medical Sciences, lot 201908057T) [17,18] was used for immunization. Each mouse was immunized via the intraperitoneal (I.P.) route at a dose (IMD) of 0.3 EU/50 μL per injection, with an interval of 1 week. Depending on the purpose of the experiments, one- to three-week-old mice were assigned to groups (*N* = 5), with diverse immunization times (Figure 1). Another group of mice was mock-immunized with 50 μL of adjuvant as the negative control.

### Assessment of binding and neutralizing antibodies

Sera were prepared from blood samples collected once a week from immunized or unimmunized mice via the medial canthus. The binding antibodies against EV-A71-VP1 were detected as described previously [29]. Briefly, 96-well plates were coated with recombinant protein, and blocked with 1% BSA (w/v) (A8010, Solar Bio, Beijing, China). Diluted sera were added to the wells, and a goat anti-mouse IgG H&L (HRP) (ab6789, Abcam, United Kingdom) was used as the secondary antibody. TMB substrate solution (Tetramethylbenzidine Single-Component Substrate solution, SE1005, Korad Bio, Beijing, China) was used for detection, followed by the addition of stop solution (C1058, Solarbio, Beijing, China), after which the absorbance at 450 nm was recorded using a SpectraMax i3x microplate reader (Molecular Device, San Jose, USA). Neutralizing antibodies against EV-A71 virus were detected using a previously described neutralization assay [22]. Briefly, sera were diluted in a gradient, mixed with virions of EV-A71 Isehara strain in 96-well plates and incubated for 2 h. Then, a suspension of human rhabdomyosarcoma cells (RD) was added and cultured for 7 days. The neutralizing antibody titre was defined as the reciprocal of the maximum dilution that inhibited cytopathic changes by 50% [42]. The same virus strain was used in the neutralization test and the *in vivo* challenge experiments.

### Virus and challenge

Human rhabdomyosarcoma (RD) cells were obtained from the Center for Cell Resource Conservation Research, NIFDC. The cells were maintained in Dulbecco’s modified Eagle’s medium (DMEM, Gibco) supplemented with 10% faetal bovine serum (FBS, Gibco) and 1% penicillin–streptomycin solution (PS, Gibco). Since our previous study had shown that EV-A71 Isehara strain (CDV-Isehara/Japan/99, GenBank NO. AB811772, kindly provided by Prof. Satoshi Koike, Tokyo Metropolitan Institute of Medical Science, Tokyo, Japan) [43] could infect hSCARB2 mice [40], it was amplified in RD cells to a titre of 1.0 × 10^8^ TCID_50_ for the challenge experiments. Immunized or unimmunized hSCARB2 mice were challenged with the virus via an 800 μL intravenous injection (I.V.) or 40 μL intracranial injection (I.C.). Control mice were injected with DMEM by the same route, with 5–10 mice in each group.

### Quantification of viral loads

The mice were sacrificed 14 days post-infection, after which tissues (brain, lungs and skeletal muscles of hind limbs) were dissected, and immediately immersed in RNAlater stabilization reagent (Takara, Shiga, Japan) for virus load quantification. TRIZOL reagent (Takara, Shiga, Japan) was used to extract the total RNA following the manufacturer’s instructions. The PrimeScript RT reagent Kit with gDNA Eraser (RR047A, Takara, Shiga, Japan) was used to convert the RNA into cDNA, which was quantified using the TB Green Premix Ex Taq II (RR820A, Takara, Shiga, Japan) on a Light Cycler 480 Real-Time PCR system, using the primers EV71-VP1-F (AAGCACTTCTGTTTCCC) and EV71-VP1-R (ATTCAGGGGCCGGAGGA), designed according to the C- and N-termini of VP1 [29,43]. The PSVA-EV71 plasmid (kindly provided by Prof. Satoshi Koike, Tokyo Metropolitan Institute of Medical Science, Tokyo, Japan) [43], containing the EV71-VP1 gene, was used as the standard. The plasmid concentration was determined using a Nanodrop2000 spectrophotometer (Thermo Fisher, USA). The PCR amplicon of the VP1 gene in the sample was compared with different concentrations of PSVA-EV71 in the standard curve.

### Immunohistochemistry

The brain, muscle, and lung tissues were fixed in 10% neutral formalin fixation solution for pathological observation of macroscopic changes and histopathological examination. The tissues were trimmed, dehydrated, embedded, sliced into sections about 3 μm thick, and stained with hematoxylin and eosin (HE). The paraffin sections were immersed in 3% hydrogen peroxide (H_2_O_2_) for 10 min to block endogenous peroxidase activity and then immersed and heated in citrate buffer (pH 6.0) for 10 min for antigen recovery. The sections were incubated with Enterovirus 71 antibody at 1:200 dilution (batch no.: GTX124261; original concentration 6.15 mg/mL; GeneTex, Inc.) as the primary antibody at 4°C overnight, followed by a goat anti-mouse/rabbit IgG working solution PV9000 (batch no.: 2014F0706; Beijing Zhongshan Golden Bridge Biotechnology Co., Ltd.) before diaminobenzidine (DAB) staining. Then, the sections were counterstained with hematoxylin, dehydrated, cleared, and covered for microscopic observation.

### Cytokine assay

Sera prepared from blood samples collected 14 days post-infection were used for the determination of cytokines using a Bio-Plex 200 system and Bio-Plex Pro Mouse Cytokine 23-plex Assay (M60009RDPD, Bio-Rad, CA, USA), according to the instruction manual (10014905, Bio-Rad, CA, USA).

### Statistical analysis

The results are expressed as the mean values with standard deviations. The data were analysed using Prism software 7.0 (GraphPad Inc, San Diego, CA, USA). The significance of differences was assessed using one-way ANOVA or multiple *t*-test. Differences with *P* < .05 were considered statistically significant.

## Results

### A high titre of neutralizing antibodies was elicited by vaccination in one-week-old suckling mice

To identify the best immunization strategy, three-week-old hSCARB2 mice were immunized at 0.5 EU/500 μL administered one, two and three times, respectively, with an interval of 1 week (Figure 1(A-a)). Serum samples were collected each week to assess the titres of binding and neutralizing antibodies. As expected, repeated immunization resulted in higher titres of both binding and neutralizing antibodies. However, the mice produced similar titres of antibodies (*P* > .05) after two or three rounds of immunization, and the titre was significantly higher than following single immunization in both cases (Figure 1(A-b, c), *P *< .0001). Since immunization with two doses 1 week apart produced a high titre of antibodies, it was selected as the optimal vaccination strategy for further experiments.

Since we planned to challenge the mice at 3–4 weeks of age, the optimal susceptible age [40,41], we further determined the earliest age at which the mice could produce sufficient antibodies. One-, two- and three-week-old mice were subjected to two vaccine injections 1 week apart (Figure 1(B-a)). We found that the three groups of mice produced high titres of binding and neutralizing antibodies at the nineth week, with no significant differences between groups (*P* > .5). At the fourth week, the one-week-old mice produced higher levels of binding (*P *< .001) and neutralizing antibodies (*P *< .05) than the others (Figure 1(B-b, c)).

These experiments showed that one-week-old hSCARB2 mice could produce high titres of binding and neutralizing antibodies at 3–4 weeks of age, which is an age at which the mice are still susceptible to EV-A71 challenge.

### Vaccination protected hSCARB2 mice from EV-A71 challenge

**Figure 2.**
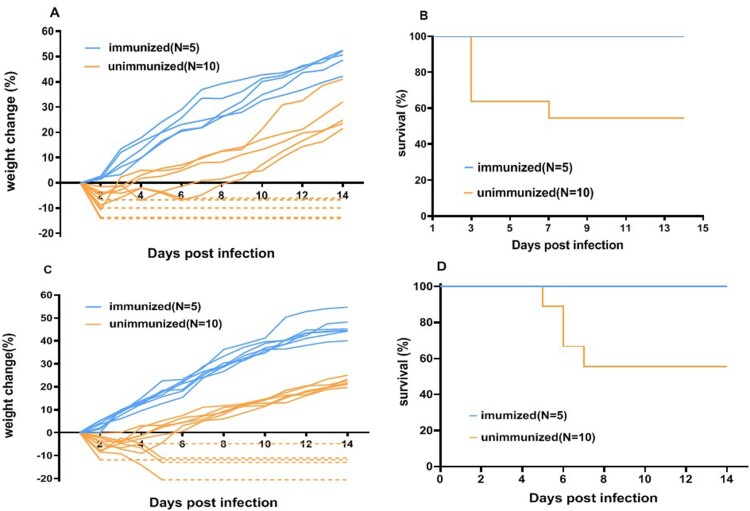
Weight change and survival of hSCARB2 mice after infection with EV-A71. One-week-old hSCARB2 mice were immunized twice with an inactivated EV-A71 vaccine via the I.P. route, with an interval of 1 week. The unimmunized mice were injected with the adjuvant. Four-week-old immunized and unimmunized mice were challenged with EV-A71 live virus (a and b) injected via the I.C. or (c and d) the I.V. route. The dotted lines represent the death of the mice. No mice died following injection with DMEM culture without virus (*N* = 5), not shown in the figure.

To investigate the effectiveness of the immunization procedure, one-week-old hSCARB2 mice were immunized two times with a marketed vaccine or adjuvant intraperitoneally (I.P.). Following 2 weeks after the second vaccination, both immunized and unimmunized mice were challenged with an intracranial (I.C.) EV-A71 injection at four weeks of age. The body weight and death rate were recorded every day (Figure 2(a,b)). All of the unimmunized mice showed severe weight loss on the first 2–3 days post-infection. While some mice gradually regained their weight, about 50% showed severe signs of infection and died (Figure 2(b)). By contrast, the immunized mice showed no weight loss during the same period, and increased their body weight until the end of the experiment, whereby no mouse died.

Considering that intracranial challenge may cause accidental brain damage and death, we carried out another experiment using an intravenous (I.V.) challenge. Almost all the mice in the unimmunized group lost weight and 5 out of 11 died at 2–3 days post-infection (Figure 2(c,d)). As expected, no deaths occurred in the immunized mice. These results indicated that vaccination could protect mice from a fatal challenge. Additionally, the results confirmed that the hSCARB2 mice can be infected with EV-A71 via different routes, at the age of 3–4 weeks.

### The immunized mice showed alleviated clinical symptoms

**Figure 3.**
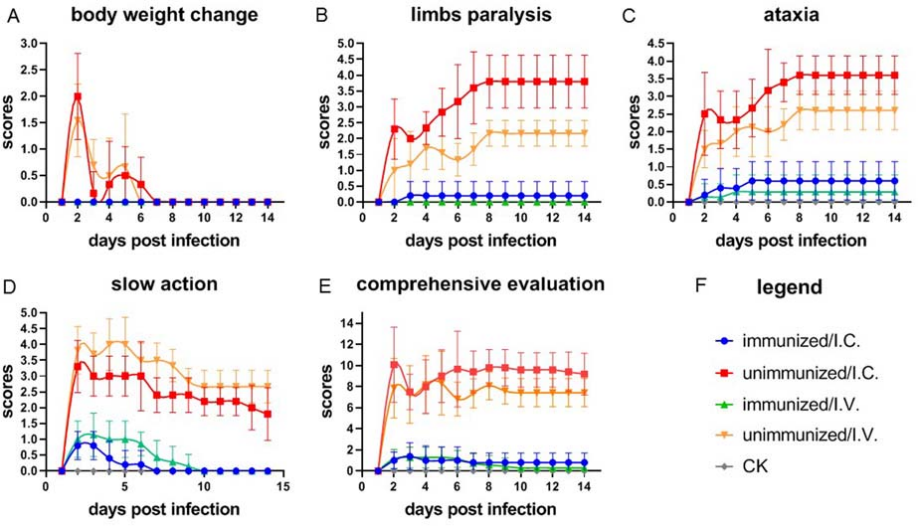
Clinical scores of hSCARB2 mice infected with EV-A71. Four-week-old immunized and unimmunized mice were challenged with EV-A71 live virus via the I.C. or I.V. route. As a negative control, four-week-old mice were injected with DMEM, *N* = 5. The four clinical symptoms were observed each day after the mice were infected, and scored followed the criteria described in Table 1. (a) body weight change, (b) limbs paralysis, (c) ataxia, (d) slow action, and (e) comprehensive evaluation of the four clinical symptoms. The data represent the mean scores obtained from the indicated number of mice per group.

To further evaluate the protective effect of the vaccine on the mice, we assessed the loss of weight, limb paralysis, slow action, and ataxia as clinical symptoms to quantify the severity of illness. The scoring criteria are shown in Table 1, with higher scores indicating more severe symptoms. The mice were scored each day after the challenge (Figure 3).

**Table 1.** Criteria for the clinical symptom scoring of mice challenged with EV-A71 live virus.

Score	0	1	2	3	4	5
Weight loss	Normal	Less than 5%	5–10%	10–15%	15–20%	More than 20%
Limb paralysis	Normal	Weak	Moderate	Serious	One limb paralysis	Planovalgus
Ataxia	Normal	Slightly	Unbalanced	Shivering	Deflection walk	Shaking
Slow action	Normal	Slightly	Low reaction	Moderate	Moderately severe	Immobility

The unimmunized mice, challenged via the I.C. or I.V. route, showed significant loss of weight, especially two days post-infection (Figure 3(a)). The surviving mice regained the weight corresponding to the control group by day 7. Neurological symptoms caused by EV-A71 infection have been reported in clinical studies [44] and mouse models [45]. In this study, tetraplegia was observed in the unimmunized group, mainly occurring in the back limbs and occasionally in the forelimbs. Limb paralysis was more severe in the I.C. challenge group than in the I.V. group, indicating that the virus could enter into the brain directly, having a greater effect on the nervous system when administered via the I.C. route. By contrast, the mice challenged via the I.V. route were protected by the blood–brain barrier, which reduced the impact on the nervous system. The unimmunized mice showed symptoms of ataxia, with a similar trend to that of paralysis (Figure 3(c)).

After the challenge, all of the mice showed various degrees of slow action, whereby the symptoms of unimmunized mice were severe, with very slow remission. The immunized mice showed weak symptoms in the first few days after the challenge, and the symptoms soon subsided and disappeared.

The I.V. challenge appeared to have a greater effect on the movement of mice, possibly causing greater muscle damage than the I.C. challenge (Figure 3(d)).

When the indicators of weight loss, paralysis, tardiness, and ataxia were combined (Figure 3(e)), we also found that the score of the unimmunized group was significantly higher than that of the immunized group. Moreover, the I.C. challenge group had more severe syndromic symptoms than the I.V. challenge group, perhaps reflecting the neurotropic characteristics of this virus.

These indicators are stable and well differentiated, and can be used as clinical observation indicators to evaluate the efficacy of vaccines *in vivo* (Figure 3(a–f)).

### Immunization significantly reduced the viral loads in target organs

**Figure 4.**
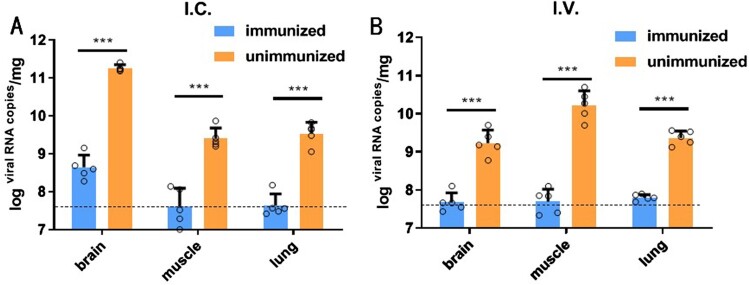
Quantification of the virus genome copy numbers in brain, muscle, and lung tissues using qPCR. The mice (*N* = 5) that were challenged with EV-A71 were euthanized at 14 days post-infection, and the brain, muscles, and lungs were collected. Total RNA was extracted from the tissues for real-time PCR amplification of the VP1 gene in the samples. The PSVA-EV71 plasmid was used to construct a standard curve with a known copy number. (a) injection via the I.C. route, (b) injection via the I.V. route. The multiple *t*-test was used for statistical analysis. ***P < .001.

To assess the effect of immunization on virus clearance in target organs, the viral loads in the brain, lungs, and muscles collected at 14 days post-infection were quantified using real-time PCR. As shown in Figure 4, large copy numbers of the viral genome were detected in the brain, muscle and lung tissues of unimmunized hSCARB2 mice, indicating that this mouse model is highly susceptible to EV-A71 infection. By contrast, the immunization remarkably reduced the viral load in the tested organs, even below the limit of detection (LOD), in both the I.C. and I.V. challenge groups (Figure 4).

### No obvious pathological changes were observed in the immunized mice

**Figure 5.**
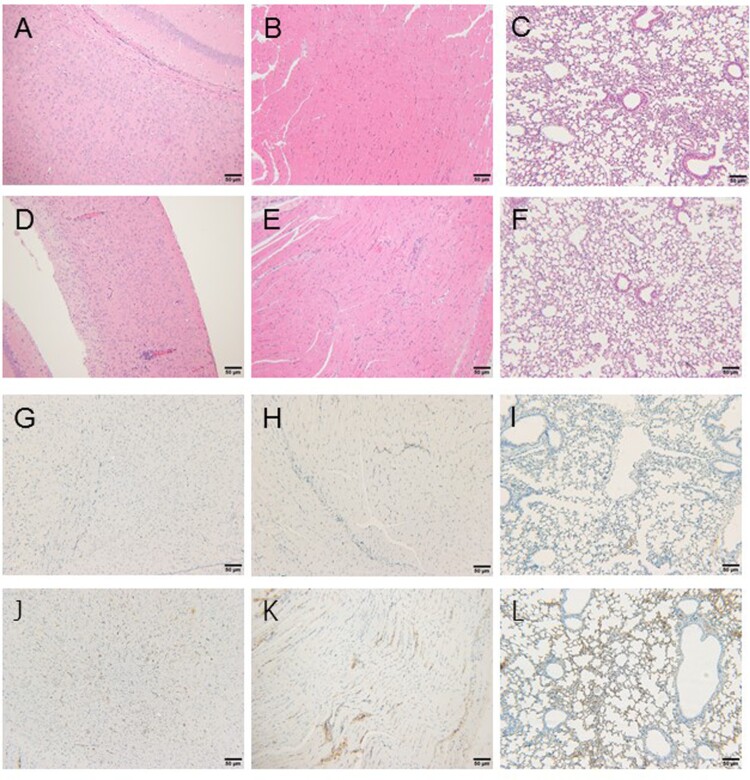
Histopathological analysis of brain, muscle, and lung tissues of mice after challenge with EV-A71 via intracerebral injection (magnification ×100). Three euthanized mice were used to examine the pathological changes in part of the tissues. (a–c) Hematoxylin and eosin staining showed no obvious histopathological changes in the brain (a), muscles, (b) and lungs (c) of mice immunized with the EV-A71 vaccine. (d–f) Hematoxylin and eosin showed minimal perivascular inflammatory infiltrates in the brain (d), minimal mixed cell infiltration in the muscle interstitium (e) and no obvious histopathological changes in the lungs (f) of unimmunized mice. (g–i) Immunohistochemistry (IHC) in tissue of immunized mice after challenged with EV-A71 via the I.C. route, with negative staining of the brain (g), muscle, (h) and lung (i) tissues. (j–l) Immunohistochemistry (IHC) in tissues of unimmunized mice after challenge with EV-A71 via the I.C. route, with positive staining of the brain (j), muscle (k), and lung (l) tissues.

Brain, muscle, and lung tissues collected from sacrificed mice at 14 days after I.C. challenge were collected for histopathological observation and virus staining (Figure 5). Perivascular inflammatory infiltrates in the brain (Figure 5(d)), and mixed cell infiltration in the muscle interstitium (Figure 5(e)) were observed in the unimmunized mice. Consistent with this observation, the immunohistochemical staining for EV-A71 virus was also positive (Figure 5(j,k)). Although no typical pulmonary pathological changes were observed (Figure 5(f)), the lungs were positive for EV-A71 virus (Figure 5(l)). By contrast, there were no lesions in the corresponding tissues of immunized mice (Figure 5(a–c)), and the virus staining was negative as well (Figure 5(g–i)). Analysis of the I.V. challenge group yielded similar results (data not shown).

### Vaccination prevented the upregulation of cytokines

**Figure 6.**
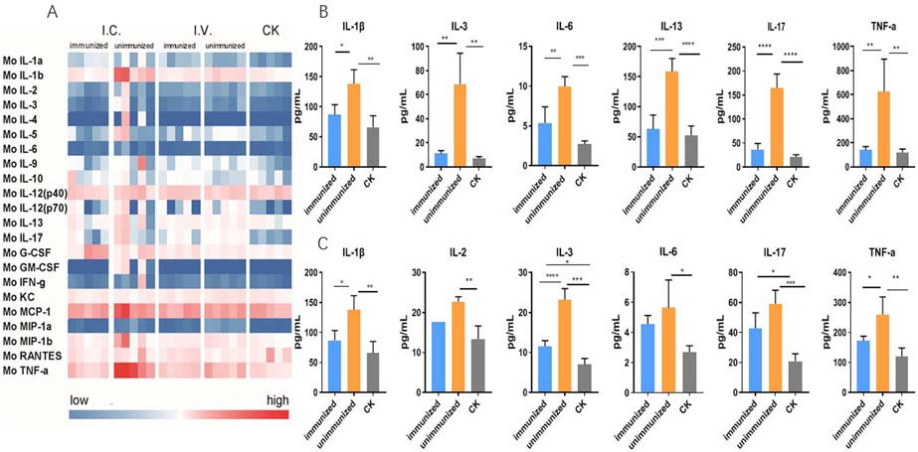
The levels of 22 cytokines in the serum of mice after challenge with EV-A71 live virus. (a) Heat map of differences among the 22 cytokines detected in the mouse serum. The differences were assessed using single-factor analysis of variance (one-way ANOVA); different colours show significant differences. The cytokines which showed significant differences were analysed. (b) IL-1β, IL-3, IL-6, IL-13, IL-17, and TNF-α showed significant differences when challenged via the I.C. route, while (c) IL-1β, IL-2, IL-3, IL-6, IL-17, and TNF-α showed significant differences when challenged via the I.V. route. One-way ANOVA was used for statistical analysis and the error bar of each group was included. *P < .05, **P < .01, ***P < .001, and ****P < .0001.

Sera were collected from both immunized and unimmunized mice 2 weeks post-challenge for cytokine analysis. According to the heat map, the cytokine profile of the unimmunized group changed greatly following the viral challenge, while that of the immunized group changed only slightly, presenting a similar profile to the control (Figure 6(a)). The expression of IL-1β, IL-3, IL-6, IL-13, IL-17, and TNF-α was significantly upregulated in the mice challenged via the I.C. route (Figure 6(b)), while in the I.V. challenged group, the expression of IL-1β, IL-2, IL-3, IL-13, IL-17, and TNF-α was markedly increased (Figure 6(c)). IL-1β, IL-3, IL-13, IL-17, and TNF-α were upregulated in both groups, which was expected since these cytokines are common signals of an inflammatory response after EV-A71 infection.

## Discussion

To improve vaccine safety, or prevent the spread of changed dominant epidemic strains, new types of vaccines, such as the recombinant protein vaccines and the EV-A71/Cox-A16 combination vaccine, are under development [46,47]. Therefore, it is still necessary to establish an efficient and accurate *in vivo* efficacy evaluation method, even though EV-A71 vaccines are already on the market. However, the current evaluation assay is based on transferred maternal antibodies [28], which makes it challenging to conduct and can lead to inaccurate results. In our opinion, this indirect evaluation method had to be adapted because of the lack of animal models that could maintain susceptibility at an older age. The age of mice has a strong influence on the susceptibility to enteroviruses infection [26], and the wild-type mice could not meet the requirements of maintaining susceptibility until vaccine-induced antibody production.

It is necessary to establish animal models that remain susceptible at older weeks of age. Scavenger receptor class B member 2 is the main cellular receptor of EV-A71 in humans [34]. Accordingly, two transgenic mouse lines expressing this receptor showed a longer duration of susceptibility, to 2 [38] or 6 weeks of age [37], but they showed no major symptoms of HFMD. Using stem-cell targeting technology, we established an hSCARB2 knock-in mouse model [40] that was highly susceptible at 3–4 weeks of age, showing weight loss (Figure 3), viral replication, and typical clinical symptoms, leading to death, and remained susceptible at 5–6 weeks of age [41,42]. This mouse model laid a foundation for the establishment of new methods for evaluating the efficacy of vaccines *in vivo*.

Despite having an appropriate mouse model, the existing immunization procedures, such as selecting mice at 4–6 weeks of age for initial vaccination [48], are an obstacle to the development of new methods for vaccine evaluation. It is generally believed that the immune systems of infants and suckling mice are insufficiently developed, having a weak response to innate stimuli, the poor immune-stimulating activity of antigen-presenting cells, and only a limited adaptive lymphocyte response, resulting in weak immune memory and an ineffective vaccine response [49,50]. In order to overcome the disadvantages of the existing evaluation methods, we designed a novel immunization procedure. Firstly, we found that two rounds of immunization could elicit sufficient titres of binding and neutralizing antibodies (Figure 1). Secondly, we intended to challenge the hSCARB2 mice at 3–4 weeks of age, the optimal susceptible age for EV-A71 infection. Thirdly, the production of antibodies requires 2 weeks based on our experiments. Consequently, we decided to select one-week-old suckling hSCARB2 knock-in mice for immunization via two doses 1 week apart. Incidentally, high titres of binding and neutralizing antibodies were produced at 4 weeks (Figure 1), which protected the mice against a lethal challenge with EV-A71 live virus (Figures 2–4). This improved immunization strategy not only enabled the establishment of the new method, but also raised an interesting question. Different from traditional understanding, it appears that one-week-old mice also have a sufficiently developed immune response, which enables them to produce antibodies. Whether this is a unique characteristic of hSCARB2 knock-in mice or a general pattern needs further study. It may, therefore, be fruitful to test more vaccines in other transgenic or wild-type mice at this age.

Based on the susceptible hSCARB2 knock-in model, together with the new immunization strategy, we successfully established a new method to directly evaluate the efficacy of EV-A71 vaccines *in vivo*. This method does not rely on maternal antibodies which makes it simple, accurate and easy to operate, with a confirmed evaluation index. The body weight, clinical symptoms, viral load, pathological changes, and upregulation of cytokines can be used as evaluation indicators, alone or in combination, according to different requirements. Ayumi et al. described a similar method of evaluating the efficacy of EV-A71 *in vivo* [21] using a transgenic mouse expressing hSCARB2, but a traditional vaccination procedure was used, and the test depends on a high-dose challenge with a virulent strain.

Studies on the pathological mechanisms of EV-A71 revealed that many cytokines, such as TNF-α, IFN-γ, IL-10, IL-22, IL-17F, IL-35, IL-8, IL-1β, IL-33, IL-4, IL-13, IL-6, IL-23, MCP-1, G-CSF, and IP-10, are associated with severe infection [45,51]. In our study, we found that virus infection caused the upregulation of multiple cytokines in the hSCARB2 mouse model. Five cytokines, including IL-1β, IL-3, IL-6, IL-13, IL-17, and TNF-γ, were upregulated in both the I.C. and I.V. challenge groups (Figure 6). It has been reported that high levels of cytokines such as IL-1β, IL-6, IL-10, and IFN-γ are correlated with clinical severity in patients with pulmonary oedema and encephalitis [52,53], indicating that the immune response of the hSCARB2 mouse model was similar to that of humans. Vaccination prevented the increase in the expression of these cytokines, keeping them at normal levels, indicating that these cytokine responses might be directly related to vaccine-induced immunity.

In this study, only one EV-A71 strain was tested, which is not representative of the full epidemiological situation. Thus, more strains should be tested using this model in the future. However, the hSCARB2 knock-in mouse model is not susceptible to Cox-A10 or A16, so more animal models should be developed in the future.

In conclusion, we established a practical direct assay to evaluate the efficacy of EV-A71 vaccines *in vivo* based on the hSCARB2 model mice. The evaluation results are more accurate because this method is independent of transferred maternal antibodies. It can be used in the development of new vaccines as well as the quality control of licensed vaccines.
